# Supplementary material for: Optimization of the Decolorization of the Reactive Black 5 by a Laccase-like Active Cell-Free Supernatant from Coriolopsis gallica
Source: Microorganisms. 2022 May 31;10(6):1137. doi: 10.3390/microorganisms10061137 (PMC9227205; doi:10.3390/microorganisms10061137)
Supplement: Supplementary file 1 [file microorganisms-10-01137-s001.zip › microorganisms-1711812-supplementary.pptx]

## Slide 1
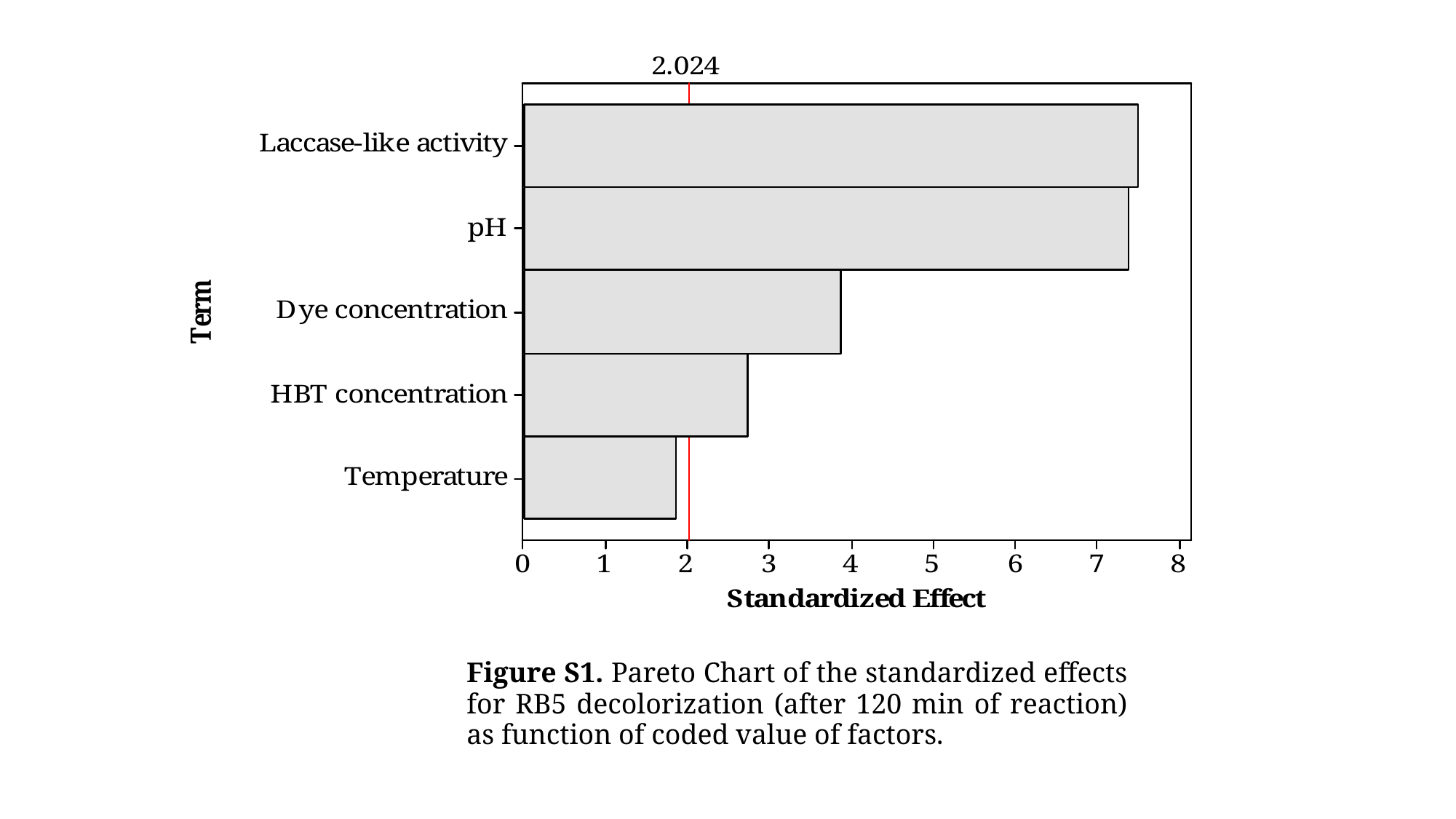

Figure S1. Pareto Chart of the standardized effects for RB5 decolorization (after 120 min of reaction) as function of coded value of factors.

## Slide 2
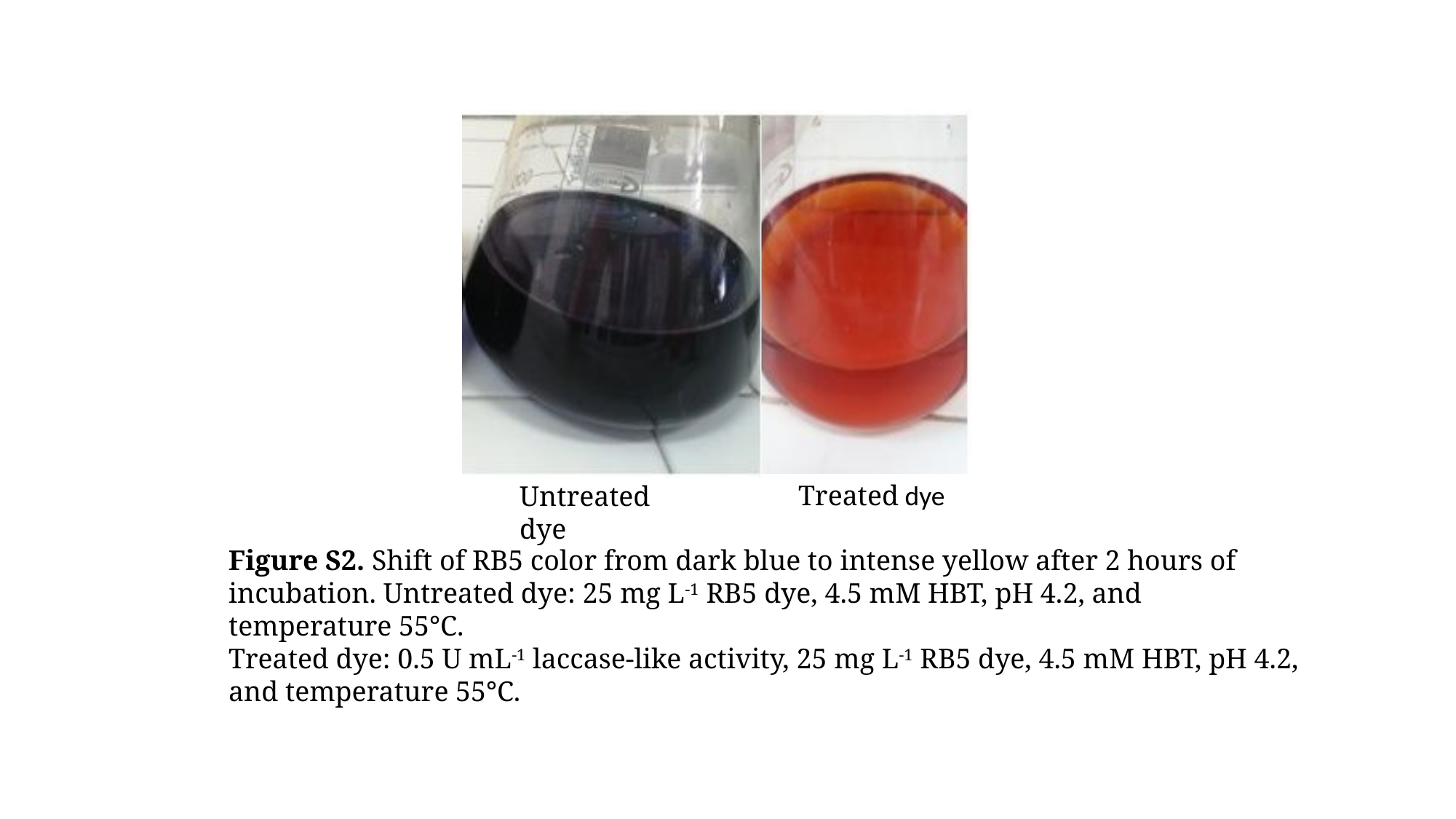

Treated dye
Untreated dye
Figure S2. Shift of RB5 color from dark blue to intense yellow after 2 hours of incubation. Untreated dye: 25 mg L-1 RB5 dye, 4.5 mM HBT, pH 4.2, and temperature 55°C.
Treated dye: 0.5 U mL-1 laccase-like activity, 25 mg L-1 RB5 dye, 4.5 mM HBT, pH 4.2, and temperature 55°C.

## Slide 3
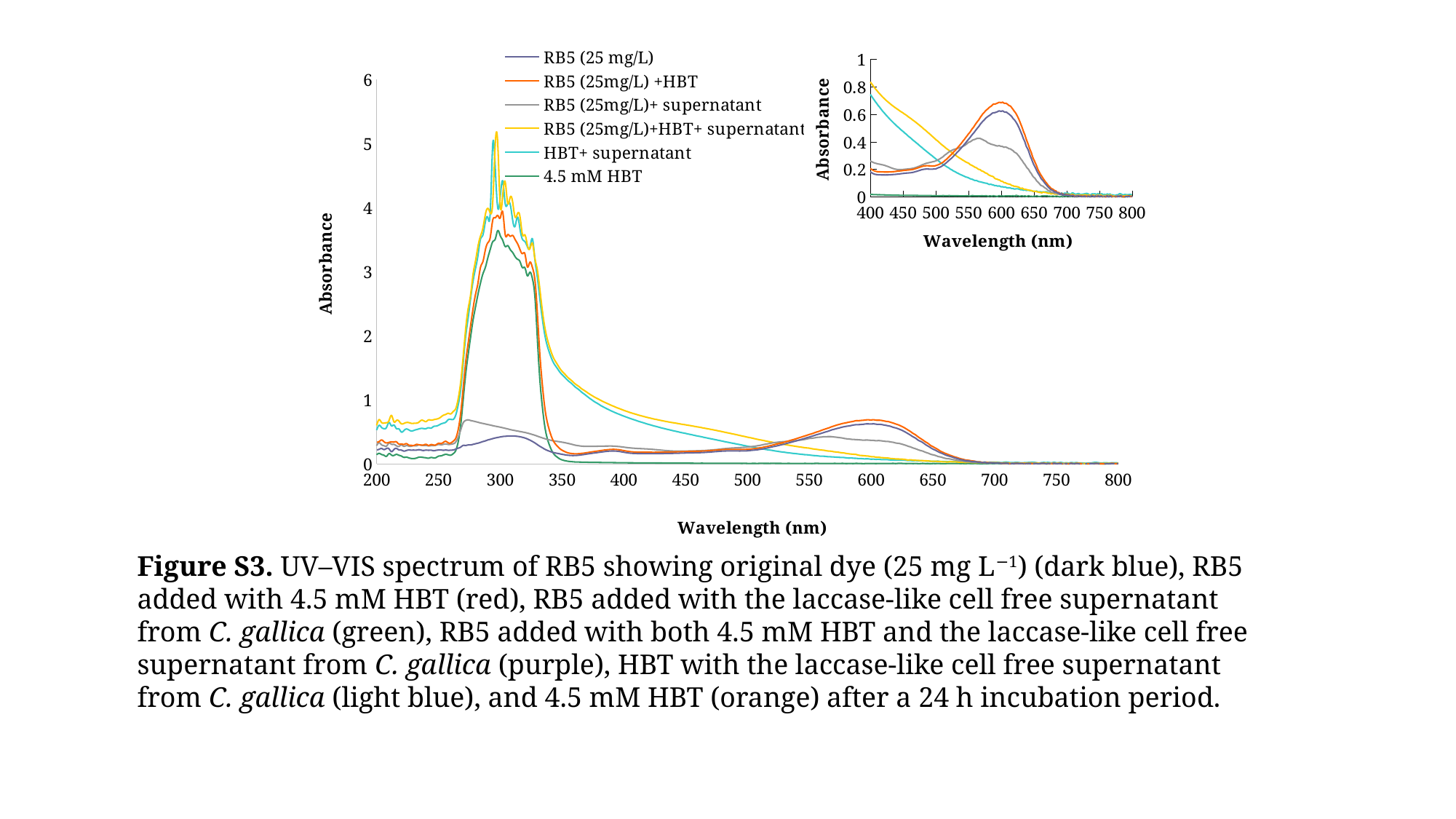

### Chart
| Category | RB5 (25 mg/L) | RB5 (25mg/L) +HBT | RB5 (25mg/L)+ supernatant | RB5 (25mg/L)+HBT+ supernatant | HBT+ supernatant | 4.5 mM HBT |
|---|---|---|---|---|---|---|
### Chart
| Category | RB5 (25 mg/L) | RB5 (25mg/L) +HBT | RB5 (25mg/L)+ Laccase | RB5 (25mg/L)+HBT+ Laccase | HBT+ Laccase | 4.5 mM HBT |
|---|---|---|---|---|---|---|Figure S3. UV–VIS spectrum of RB5 showing original dye (25 mg L−1) (dark blue), RB5 added with 4.5 mM HBT (red), RB5 added with the laccase-like cell free supernatant from C. gallica (green), RB5 added with both 4.5 mM HBT and the laccase-like cell free supernatant from C. gallica (purple), HBT with the laccase-like cell free supernatant from C. gallica (light blue), and 4.5 mM HBT (orange) after a 24 h incubation period.

## Slide 4
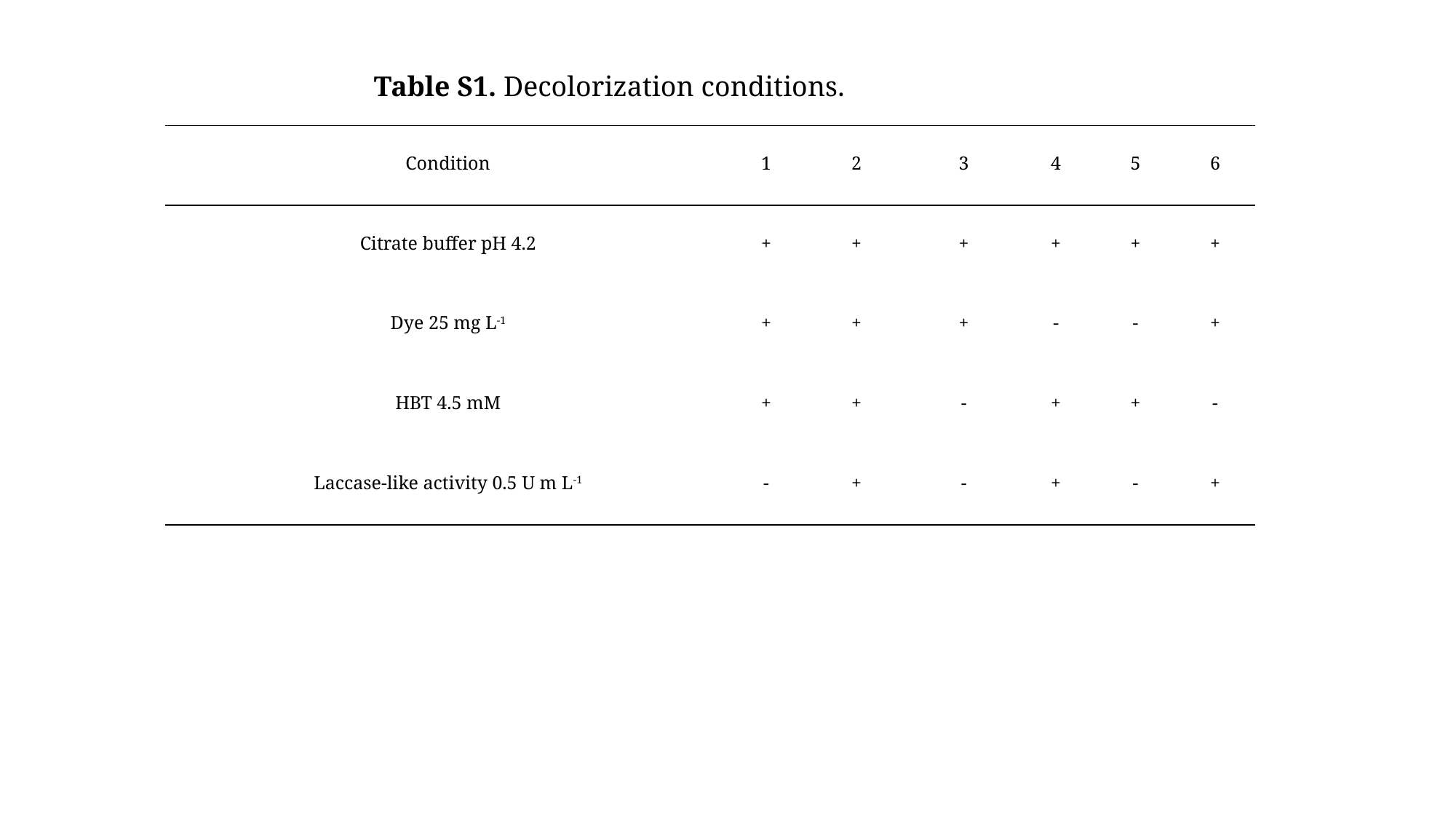

Table S1. Decolorization conditions.
| Condition | 1 | 2 | 3 | 4 | 5 | 6 |
| --- | --- | --- | --- | --- | --- | --- |
| Citrate buffer pH 4.2 | + | + | + | + | + | + |
| Dye 25 mg L-1 | + | + | + | - | - | + |
| HBT 4.5 mM | + | + | - | + | + | - |
| Laccase-like activity 0.5 U m L-1 | - | + | - | + | - | + |
